# GC–MS Characterization and Pharmacological Activities of Natural Products from *Vitex agnus-castus*

**DOI:** 10.3390/biomedicines14061365

**Published:** 2026-06-17

**Authors:** Ibrahim M. Aziz, Rawan M. Alshalan, Amal Saad Al-Shenifi, Fuad Alanazi, Abdulhadi M. Abdulwahed, Amal Khalaf Alghamdi, Sahar Abdulaziz AlSedairy

**Affiliations:** 1 Department of Botany and Microbiology, College of Science, King Saud University, P.O. Box 2455, Riyadh 11451, Saudi Arabia; ralshalaan@ksu.edu.sa (R.M.A.); aalshenifi@ksu.edu.sa (A.S.A.-S.); ahamdan1@ksu.edu.sa (A.K.A.); 2 Department of Clinical Laboratory Sciences, College of Applied Medical Sciences, King Saud University, Riyadh 12372, Saudi Arabia; foalanazi@ksu.edu.sa (F.A.); aabdulwahed@ksu.edu.sa (A.M.A.); 3 Department of Food Sciences and Nutrition, College of Food and Agricultural Sciences, King Saud University, P.O. Box 2460, Riyadh 11451, Saudi Arabia; ssudairy@ksu.edu.sa

**Keywords:** natural products, bioactive compounds, pharmacological activity, infectious diseases

## Abstract

**Background/Objectives**: *Vitex agnus-castus* L. is a well-known medicinal herb shown to be effective in treating gynecological disorders. However, no systematic comparative studies have been conducted between *V. agnus-castus* leaf extract (VACLE) and *Vitex agnus-castus* seed extract (VACSE). We performed gas chromatography–mass spectrometry (GC–MS) analysis of VACLE and VACSE and measured total phenolic and flavonoid contents. **Methods**: The bioactivity testing included antioxidants, antibacterial, antifungal, anticancer, and antidiabetic activities. **Results**: A total of 55 GC–MS compounds were identified in VACLE and 34 in VACSE; isoamyl formate (27.96%) and a pyranone derivative (14.11%) were detected exclusively in VACLE, whereas cis-linoleic acid (40.58%) and palmitic acid (21.87%) predominated in VACSE. VACLE showed significantly higher TPC (94.12 vs. 54.12 mg GAE/g DW) and TFC (82.00 vs. 42.00 mg QE/g DW). The VACLE demonstrated moderate antioxidant activity and generally stronger bioactivity than VACSE, as evidenced by its lower ABTS^+^ radical scavenging IC_50_ value (55 vs. 70 μg/mL), antibacterial activity (MIC: 6.25–50 vs. 12.5–100 μg/mL), anticancer activity against HepG2 cells (IC_50_: 93.2 vs. 247.5 μg/mL), and antidiabetic activity through α-amylase inhibition (IC_50_: 28.7 vs. 70.1 μg/mL). VACSE exhibited greater antifungal activity than VACLE against all tested *Candida* strains, with the highest activity observed against *C. parapsilosis* (MIC: 6.25 ± 2.26 μg/mL). VACLE induced transcriptional changes consistent with caspase-mediated apoptosis, characterized by increased expression of caspase-8, caspase-9, and Bax and decreased expression of Bcl-2/Bcl-xL, pending protein-level confirmation. **Conclusions**: In conclusion, VACLE exhibits notable antioxidant, antibacterial, anticancer, and antidiabetic properties, whereas VACSE shows greater antifungal activity. These findings highlight tissue-specific differences in phytochemical composition and *in vitro* biological activities and provide a basis for future studies involving compound isolation, mechanistic validation, toxicity assessment, and *in vivo* evaluation.

## 1. Introduction

*Vitex agnus-castus* L. (*Lamiaceae*), often known as chaste tree or monk’s pepper, is a perennial shrub native to the Mediterranean and Central Asian regions. The fruits (seeds) were used in folk medicine to treat gynecological disorders such as premenstrual syndrome, cyclical mastalgia, hyperprolactinemia, and menopausal disorders [1,2]. Current pharmacological studies have confirmed the use of its extracts to inhibit prolactin secretion via stimulation of dopaminergic D2 receptors, primarily due to the presence of diterpenes such as rotundifuran and vitexilactone, as well as triterpenes such as 3-epi-maslinic acid [3,4,5]. Furthermore, the herb is well known for its antioxidant, anti-inflammatory, antibacterial, and anticancer properties, owing to its high flavonoid content (casticin, orientin, vitexin) and phenolic acid content [6,7]; however, the differential distribution of these metabolites and their relationship to bioactivity have not yet been clearly elucidated. While there have been independent studies on the leaf or the seed extracts of *V. agnus-castus*, there is no study that compares the efficacy of leaves and seeds in identical biochemical assays. Such information will be critical in deciding on which plant part can be used for which purpose.

Ecologically and metabolically, the differential accumulation of secondary metabolites between leaves and seeds represents different biological roles for each organ. Due to their exposure to environmental stresses such as ultraviolet radiation and insect herbivores, leaves usually contain larger amounts of phenolic and volatile terpenoid substances that act as defenses against these challenges [8]. Seeds, on the other hand, consist of high-energy molecules such as fatty acids and sterols that help initiate germination and early growth. They also possess defense mechanisms against seed predators [9].

The distinct traditional uses of leaves and seeds indicate that their phytochemical compositions and bioactivity profiles differ significantly; however, no comprehensive study has compared the two plant organs. The identification of tissue-specific chemical compositions and biological activities is critical for confirming ethnopharmacological claims regarding leaf extracts and determining the most suitable plant parts for specific therapeutic applications. In addition, such investigations may help prevent the substitution of leaves for seeds in commercial products and facilitate the identification of novel bioactive compounds unique to each organ. As a result, this research will present a comprehensive comparison of *V. agnus-castus* leaf extract (VACLE) and *V. agnus-castus* seed extract (VACSE). The current study’s specific goals include characterizing the gas chromatography–mass spectrometry (GC-MS) phytochemical profiles of both extracts and evaluating their antioxidative, antimicrobial, anticancer, and antidiabetic properties. GC–MS was chosen as the main analysis method because of its high resolution, reproducibility, and spectral library for both volatile and semi-volatile compounds. It is especially suitable for investigating terpenoid and fatty acid profiles in *Vitex* spp.; however, we should also acknowledge its limitations with respect to nonvolatile, polar components, such as flavonoid glycosides. To partially overcome that limitation, we add analyses of total phenolic content (TPC) and total flavonoid content (TFC).

The selection of anticancer and antidiabetic bioassays in this study was based on both the traditional medicinal uses of *V. agnus-castus* and growing evidence of these activities in members of the genus *Vitex*. Previous studies have reported the cytotoxic effects of *V. agnus-castus* seed extracts against gastric cancer cells [10] and their hypoglycemic activity in experimental animal models [11,12]. However, comparative investigations of leaf and seed extracts under identical experimental conditions remain limited. The present study was designed to address this gap by evaluating and comparing the biological activities of extracts derived from different plant organs. In addition, the phytochemical constituents commonly reported in *V. agnus-castus*, including phenolics, flavonoids, terpenoids, and fatty acid derivatives, have been associated with antioxidant, antimicrobial, anticancer, and antidiabetic properties [7].

Since oxidative stress plays a significant role in the pathogenesis of cancer, microbial infections, and metabolic disorders, the integrated assessment of these bioactivities provides a comprehensive approach for evaluating the therapeutic potential of the species. Therefore, this study aimed to characterize the GC–MS phytochemical profiles of leaf and seed extracts of *V. agnus-castus* and to compare their antioxidant, antimicrobial, anticancer, and antidiabetic activities, to identify organ-specific bioactive compounds and potential pharmacological applications.

## 2. Materials and Methods

### 2.1. Preparation of Methanolic Leaf and Seeds of V. agnus-castus

Leaves and seeds of *V. agnus-castus* L. were collected from naturally growing plants in the Al-Kharj region, Saudi Arabia, during the flowering season. Plant identity was authenticated at the Herbarium of the Department of Botany and Microbiology, College of Science, King Saud University, where a voucher specimen (KSU No. 343341) was deposited. Plant materials were collected from three individual plants to provide biological replication. The collected leaves and seeds were thoroughly washed with distilled water, air-dried at room temperature (25 ± 2 °C) in a well-ventilated, shaded environment protected from direct sunlight, and subsequently ground into a fine powder using a Pulverisette 14 mill (Fritsch GmbH, Idar-Oberstein-Georg-Weierbach, Germany) equipped with a 0.5 mm sieve.

For extraction, 10 g of powdered leaf or seed material was macerated separately in 100 mL of absolute methanol in sealed amber glass containers and maintained at room temperature with continuous agitation for 48 h. Methanol was selected because of its high polarity and extraction efficiency toward a broad spectrum of secondary metabolites, including phenolics, flavonoids, terpenoids, lipids, and fatty acids, many of which have previously been reported in *V. agnus-castus* [2]. Its widespread use in phytochemical studies facilitates the recovery of both moderately polar and polar bioactive compounds [13]. Following extraction, mixtures were filtered through Whatman No. 1 filter paper, and the filtrates were concentrated under reduced pressure using a rotary vacuum evaporator (BO410, Yamato Scientific Co., Ltd., Tokyo, Japan) at temperatures not exceeding 40 °C. The dried extracts were stored in airtight containers at 4 °C until further analysis.

The extraction yield was calculated according to the following Equation [14]:

Extraction yield (%) = (Weight of dried extract/Weight of dried plant material) × 100

### 2.2. Determination of Bioactive Components

Qualitative and quantitative analyses of VACLE and VACSE were performed using a GC–MS system (Agilent Technologies, Santa Clara, CA, USA) equipped with a split/splitless injector and a DB-5MS fused silica capillary column (30 m × 0.25 mm i.d., 0.25 μm film thickness). Helium was used as the carrier gas at a constant flow rate of 1.0 mL/min. The oven temperature program was set as follows: initial temperature at 40 °C (held for 3 min), ramped at 7.5 °C/min to 280 °C (held for 5 min), and finally increased to 290 °C (held for 1 min). The injector and detector temperatures were maintained at 250 °C and 300 °C, respectively. Samples (1 μL) were injected into split mode (1:10). Mass spectrometric detection was carried out in electron impact (EI) mode at 70 eV. The ion source and interface temperatures were set to 230 °C and 280 °C, respectively, with the quadrupole temperature set to 150 °C. Data acquisition was performed in full-scan mode over the *m*/*z* range of 40–500, with a solvent delay of 8 min. The total running time was 60 min. Compound identification was achieved by comparing mass spectra against the Wiley 275 library and the NIST libraries, using similarity indices above 90% for reliable identification.

According to the Metabolomics Standards Initiative (MSI) criteria [15], chemical identifications were assigned the following confidence levels: Level 1 (identified compound): confirmed by matching retention time and mass spectrum with an authentic standard (none in this study); Level 2 (putative identification): mass spectrum matches library spectrum with similarity ≥ 90% and linear retention index (RI) matches literature or predicted RI within ±20 units; Level 3 (tentative identification): mass spectrum matches library spectrum with similarity ≥ 90% but RI data unavailable or inconclusive; Level 4 (unknown): unknown. 

### 2.3. Determination of TPC and TFC

TPC of VACLE and VACSE was determined using the Folin–Ciocalteu method as described by Wolfe and Liu [16], with slight modifications. Briefly, 0.1 mL of plant extract (1 mg/mL) was mixed with 3 mL of distilled water. After 5 min, 2 mL of 20% (*w*/*v*) sodium carbonate (Na_2_CO_3_) was added. The reaction mixture was incubated in the dark at 25 °C for 30 min. The absorbance was measured at 720 nm using a spectrophotometer (Thermo Scientific Multiskan Sky, Waltham, MA, USA). Gallic acid (25–200 mg/mL) was used as the reference standard for calibration, and TPC was expressed as milligrams of gallic acid equivalents per gram of dry weight (mg GAE/g DW). TPC was quantified using gallic acid calibration curves with regression equations of y = 0.008x + 0.176 (R^2^ = 0.992) for VACLE and y = 0.002x + 0.0265 (R^2^ = 0.9971) for VACSE, demonstrating excellent linearity across the tested concentration range.

TFC of the VACLE and VACSE was determined using the aluminum chloride (AlCl_3_) colorimetric method described by Ordonez, Gomez et al. [17], with slight modifications. Briefly, 500 μL of VACLE and VACSE (2 mg/mL) were separately mixed with 1 mL of 2% (*w*/*v*) AlCl_3_ solution and 3 mL of sodium acetate solution (50 g/L). Subsequently, two drops of acetic acid were added, and the reaction mixture was incubated in the dark at 25 °C for 60 min. The absorbance was measured at 420 nm using a spectrophotometer (Thermo Scientific, Multiskan-Sky, Waltham, MA, USA). Quercetin was used as the calibration standard, and the results were expressed as milligrams of quercetin equivalents per gram of dry weight (mg QE/g DW). For TFC determination, quercetin was used as the external standard, and the results were expressed as mg quercetin equivalents per gram of dry weight (mg QE/g DW). Calibration curves for TPC analyses were constructed using standard solutions at concentrations ranging from 25 to 200 mg/mL. Calibration curves for TPC analysis were constructed using gallic acid standard solutions at concentrations ranging from 25 to 200 μg/mL. The calibration curves showed good linearity, with regression equations of y = 0.008x + 0.176 (R^2^ = 0.9920) for VACLE and y = 0.002x + 0.0265 (R^2^ = 0.9971) for VACSE.

### 2.4. Antibacterial Activity

#### 2.4.1. Agar Diffusion Method

The antimicrobial activities of VACLE and VACSE were evaluated against a panel of microorganisms, including Gram-positive bacteria (*Staphylococcus aureus* ATCC 29213, Enterococcus faecalis (ATCC 29212), and *Bacillus subtilis* (ATCC 23857)), Gram-negative bacteria (*Escherichia coli* (ATCC 25922), *Klebsiella pneumoniae* (ATCC 13883), and *Pseudomonas aeruginosa* (ATCC 27853)), and pathogenic fungi (*Candida albicans* (ATCC 10231), *Candida glabrata* (ATCC 2001), and *Candida parapsilosis* (ATCC 22019)).

Microbial inocula were prepared in Mueller–Hinton broth (MHB) for bacteria and Sabouraud dextrose broth (SDB) for fungi, then incubated at 37 °C for 18–24 h. The turbidity of the cultures was adjusted to 0.5 McFarland standard (≈1.5 × 10^8^ CFU/mL).

The antimicrobial activity was assessed using the agar well diffusion method with slight modifications of previously reported protocols [18,19]. Briefly, sterile Mueller–Hinton agar (MHA) plates (for bacteria) and potato dextrose agar (PDA) plates (for fungi) were inoculated with 100 µL of standardized microbial suspensions. Wells (6 mm diameter) were aseptically bored into the agar and filled with 100 µL of VACLE or VACSE at concentrations of 100–800 µg/mL, prepared in nutrient broth.

Chloramphenicol (25 µg/mL) and fluconazole (25 µg/mL) were used as positive controls for antibacterial and antifungal activities, respectively, whereas 0.01% DMSO in the corresponding culture medium served as the negative (solvent) control. Plates were allowed to pre-diffuse at room temperature for 30 min and then incubated at 37 °C for 24 h. The antimicrobial activity was evaluated by measuring the diameter of zones of inhibition (ZoI) (mm) around each well [20].

#### 2.4.2. Determination of Minimum Inhibitory Concentration (MIC), Minimum Bactericidal Concentration (MBC), and Minimum Fungicidal Concentration (MFC)

The MIC, MBC, and MFC of VACLE and VACSE were determined using the broth microdilution method with slight modifications based on the Triphenyltetrazolium chloride (TTC) assay [21].

The assay was performed in sterile 96-well microplates using MHB for bacteria and SDB for fungi. Briefly, 100 µL of sterile broth was added to each well, followed by two-fold serial dilutions of VACLE and VACSE to obtain final concentrations ranging from 1.95 to 800 µg/mL. Subsequently, 10 µL of the microbial suspension adjusted to approximately 1 × 10^6^ CFU/mL, was inoculated into each well. The microplates were incubated at 37 °C for 24 h. Following incubation, 20 µL of TTC solution (2 mg/mL in phosphate-buffered saline) was added to each well and incubated for an additional 20 min at 37 °C. The formation of a pink/red color indicated microbial growth, whereas the absence of color change indicated growth inhibition. The MIC was defined as the lowest extract concentration at which no visible color change occurs. For the determination of MBC and MFC, 100 µL aliquots from wells showing no visible growth were sub-cultured onto MHA (for bacteria) or PDA (for fungi) plates and incubated at 37 °C for 24 h. The MBC or MFC was defined as the lowest extract concentration that showed inhibition of microbial growth on agar plates [22].

### 2.5. Antioxidant Activity

#### 2.5.1. Diphenyl-1-Picrylhydrazyl (DPPH) Radical Scavenging Activity

The antioxidant potential of VACLE and VACSE was evaluated using the DPPH radical-scavenging assay, as previously described by Tian et al. (2020) [23], with minor modifications. Briefly, extracts at different concentrations (100–800 µg/mL) were prepared and mixed with 2 mL of a freshly prepared 0.08 mM DPPH solution. The mixtures were vortexed and incubated in the dark at 25 °C for 30 min. Absorbance was recorded at 517 nm using a Thermo Scientific Multiskan Sky Spectrophotometer (Thermo Fisher Scientific, Waltham, MA, USA). Ascorbic acid (100–800 µg/mL) served as the positive control. The DPPH radical scavenging activity (%) was calculated. The half-maximal inhibitory concentration (IC_50_) values were calculated by constructing a concentration-dependent inhibition change curve.

#### 2.5.2. 2,2′-Azino-Bis (3-Ethylbenzothiazoline-6-Sulfonic Acid) (ABTS^+^) Assay

ABTS^+^ radical scavenging activity of VACLE and VACSE was determined following Yu et al. (2013) [24]. ABTS^+^ was generated by reacting 7 mM ABTS^+^ with 2.45 mM K_2_S_2_O_8_ in the dark for 12–16 h and diluted to an absorbance of 0.70 ± 0.02 at 734 nm. Extracts (12.5–100 µg/mL) were mixed with ABTS^+^ solution (1:1, *v*/*v*), and absorbance was measured at 734 nm using a Thermo Scientific Multiskan Sky Spectrophotometer. The ABTS^+^ % and IC_50_ readouts were calculated as described above.

### 2.6. Cytotoxicity Assay

The cytotoxic activity of VACLE and VACSE was evaluated *in vitro* using human hepatocellular carcinoma HepG2 (ATCC HB-8065) and human breast adenocarcinoma MCF-7 (ATCC HTB-22) cell lines using the MTT (3-[4,5-dimethylthiazol-2-yl]-2,5-diphenyl tetrazolium bromide) assay. These cell lines were selected because they are widely used and well-established *in vitro* models for evaluating the anticancer potential of plant-derived compounds against liver and breast cancers, respectively [25,26]. The cell lines were obtained from the Virology Research Group (VRG), College of Science, King Saud University, Riyadh, Saudi Arabia. Cells were maintained at 37 °C under standard culture conditions and routinely confirmed to be mycoplasma-free using the LookOut^®^ Mycoplasma qPCR Detection Kit (Merck, Darmstadt, Germany). The assay is based on the reduction of MTT to insoluble purple formazan crystals by mitochondrial dehydrogenases in viable cells. Cells were cultured in Dulbecco’s Modified Eagle Medium (DMEM; Gibco, Invitrogen, Carlsbad, CA, USA) supplemented with 10% fetal calf serum (FCS) and 1% penicillin–streptomycin under standard conditions (37 °C, 5% CO_2_, humidified atmosphere). Cells were seeded into 96-well plates at a density of 2 × 10^3^ cells/well (100 µL/well) and allowed to adhere overnight. Following incubation, cells were treated with VACLE and VACSE at concentrations ranging from 0 to 400 µg/mL for 24 h. Untreated cells cultured in medium containing 0.01% DMSO served as the negative (solvent) control, while cisplatin (30 µg/mL) served as the positive control. After treatment, 10 µL of MTT solution (5 mg/mL) was added to each well, and the plate was incubated for 3 h at 37 °C. The medium was carefully removed, and the resulting formazan crystals were dissolved in 100 µL dimethyl sulfoxide (DMSO). Absorbance was measured at 570 nm with a reference wavelength of 620 nm using a microplate reader (BioTek ELx808, BioTek Instruments, Winooski, VT, USA). Cell viability (%) was calculated according to the following Equation:

The cell viability (%) = [(absorbance of untreated cells (control) − absorbance of treated cell lines)/absorbance of untreated cells (control)] × 100

as described by [27]. The IC_50_ values were determined by nonlinear regression analysis using GraphPad Prism (version 5.0, La Jolla, CA, USA).

### 2.7. Gene Expression Analysis

Human HepG2 and MCF-7 cells were seeded at 4.5 × 10^5^ cells/well in 6-well plates and incubated for 24 h to allow cell attachment. Cells were subsequently treated with the IC_50_ concentrations of VACLE or VACSE for 48 h. Following treatment, cells were harvested by trypsinization, collected by centrifugation at 500× *g* for 5 min at 4 °C, and the resulting pellets were stored at −80 °C until analysis. Total RNA was extracted using the RNeasy Micro Kit (Qiagen, Hilden, Germany) according to the manufacturer’s instructions. RNA concentration and purity were determined spectrophotometrically. Complementary DNA (cDNA) was synthesized from the extracted RNA using the Wonder RT-cDNA Synthesis Kit (Euroclone, Pero, Italy) following the manufacturer’s protocol. Quantitative reverse transcription polymerase chain reaction (RT-qPCR) was performed to evaluate the expression of apoptosis-related genes, including the pro-apoptotic genes *caspase-3*, *caspase-8*, *caspase-9*, and *Bax*, and the anti-apoptotic genes Bcl-2 and Bcl-xL. Reactions were conducted in a final volume of 20 µL containing 10 µL SYBR Green Master Mix (Qiagen, Hilden, Germany), 0.5 µL of each primer (10 µM), 2 µL of cDNA template, and nuclease-free water. Amplification was carried out using a 7500 Fast Real-Time PCR System (Applied Biosystems, Waltham, MA, USA) under the following conditions: initial denaturation at 95 °C for 3 min, followed by 40 cycles of denaturation at 95 °C for 15 s and annealing/extension at 60 °C for 30 s. Melting curve analysis was performed at the end of each run to verify amplification specificity and confirm the absence of primer-dimer formation. Gene expression levels were normalized to GAPDH, the housekeeping gene, and quantified using the 2^−ΔΔCq^ method, as described [28]. Relative expression levels were calculated by comparing treated samples with untreated control cells. The primer sequences used in this study are listed in Table 1.

### 2.8. Antidiabetic Activity

#### 2.8.1. α-Amylase Inhibitory Assay

The α-amylase inhibitory activity of VACLE and VACSE was evaluated using the 3,5-dinitrosalicylic acid (DNSA) method as described before [33], with minor modifications. This assay reduces sugars released from starch by α-amylase. Briefly, the extracts were prepared at concentrations ranging from 12.50 to 200 µg/mL in phosphate buffer (0.02 M Na_2_HPO_4_/NaH_2_PO_4_ containing 0.006 M NaCl, pH 6.9). 200 µL of each extract was mixed with 200 µL of α-amylase solution (2 U/mL; Molychem) and incubated at 37 °C for 10 min. Following pre-incubation, 200 µL of 1% (*w*/*v*) starch solution was added, and the reaction mixture was further incubated at 37 °C for 3 min. The reaction was terminated by adding 200 µL of DNSA reagent (12 g sodium potassium tartrate tetrahydrate in 8 mL of 2 M NaOH and 20 mL of 96 mM DNSA solution), followed by heating at 85 °C for 10 min in a water bath. After cooling to room temperature, the reaction mixture was diluted with 5 mL of distilled water, and absorbance was measured at 540 nm using a Thermo Scientific Multiskan Sky spectrophotometer (Thermo Fisher Scientific, Waltham, MA, USA). Acarbose was used as the positive control, while a reaction mixture without extract served as the negative control. The percentage inhibition of α-amylase activity was calculated using the following Equation:

Inhibition (%) = [(absorbance of negative control) – (absorbance of sample cell lines)/absorbance of negative control] × 100

The IC_50_ Values were determined by nonlinear regression analysis using GraphPad Prism (version 5.0, La Jolla, CA, USA).

#### 2.8.2. α-Glucosidase Inhibitory Assay

The α-glucosidase inhibitory activity of VACLE and VACSE was evaluated using yeast α-glucosidase and *p*-nitrophenyl-α-D-glucopyranoside (pNPG) as the chromogenic substrate, following the previously described method [34], with minor modifications. Briefly, α-glucosidase from *Saccharomyces cerevisiae* (EC 3.2.1.20, Sigma-Aldrich, St. Louis, MO, USA) (1 U/mL) was prepared in 0.1 M phosphate buffer (pH 6.9). A reaction mixture containing 50 μL of enzyme solution, 250 μL of phosphate buffer, and 100 μL of plant extract (12.5–200 μg/mL) was pre-incubated at 37 °C for 20 min. Subsequently, 10 μL of pNPG solution (10 mM in 0.1 M phosphate buffer, pH 6.9) was added to initiate the reaction, resulting in a final substrate concentration of approximately 0.24 mM in the reaction mixture. The reaction was incubated at 37 °C for 30 min and terminated by adding 650 μL of 1 M sodium carbonate.

The release of *p*-nitrophenol was measured spectrophotometrically at 405 nm using a Thermo Scientific Multiskan Sky microplate reader (Thermo Fisher Scientific, Waltham, MA, USA). Acarbose (12.5–200 μg/mL) served as the positive control, while reaction mixtures containing all components except the plant extract were used as negative controls. Blank samples containing extract without enzyme were included to correct for background absorbance. The percentage inhibition of α-glucosidase activity was calculated according to the following Equation:

Inhibition (%) = [(absorbance of negative control) – (absorbance of sample cell lines)/absorbance of negative control] × 100

The (IC_50_) Values were determined by nonlinear regression analysis using GraphPad Prism (version 5.0, La Jolla, CA, USA).

### 2.9. Statistical Analysis

All data were analyzed using GraphPad Prism (version 5.0, La Jolla, CA, USA). Results are presented as the mean ± standard deviation (SD) of three independent biological experiments, each performed in triplicate as technical replicates. Data normality was assessed using the Shapiro–Wilk test before statistical analysis. Comparisons between two groups were performed using the unpaired Student’s *t*-test for normally distributed data, whereas the Mann–Whitney U test was used for non-normally distributed data. For multiple group comparisons, a one-way analysis of variance (ANOVA) with the least significant difference (LSD) post hoc test was used. The LSD test was chosen because it strikes an optimal compromise between statistical power and Type I error correction in the context of our planned pairwise comparisons (i.e., leaf vs. seed extract at each concentration). However, we accept that the LSD test does not account for multiple comparisons as conservatively as Tukey’s HSD or Bonferroni correction, resulting in an increased likelihood of Type I error (false positives). As a result, data indicated as statistically significant by the LSD test should be taken as exploratory and confirmed in independent replication studies. IC_50_ values were determined using nonlinear regression in GraphPad Prism and are provided with their matching 95% confidence intervals (95% CI). A *p*-value of <0.05 was considered statistically significant. However, given the exploratory character of this study, *p*-values between 0.05 and 0.01 are presented as trends rather than clear evidence.

## 3. Results

### 3.1. Extraction Yields

The extraction efficiency of the methanolic extracts was 11.12% (*w*/*w*) for VACLE and 10.65% (*w*/*w*) for VACSE, calculated relative to the dry weight of the starting material.

### 3.2. Chemical Compositions of the VACLE and VACSE

**Figure 1:**
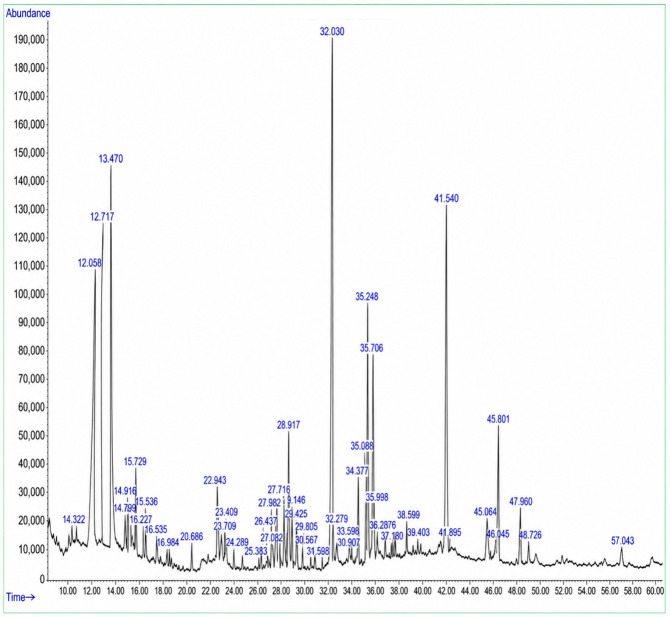
GC–MS chromatogram of VACLE showing the distribution of identified compounds. Each spectral peak corresponds to a specific phytochemical constituent, with the dominant peak representing the major compound in the extract.

**Figure 2:**
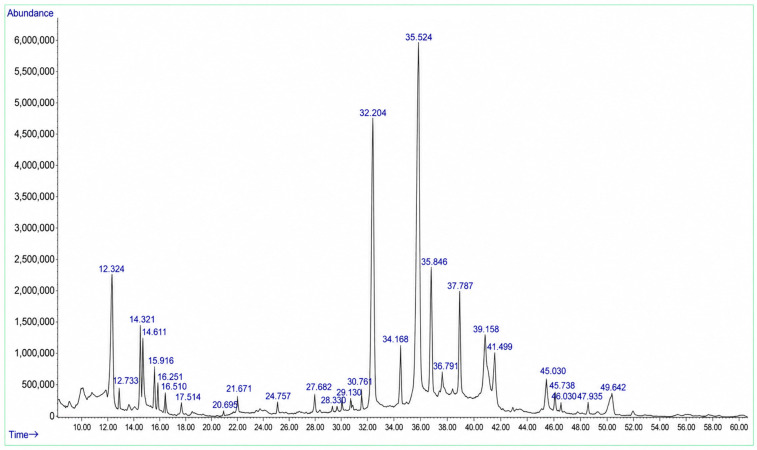
GC–MS chromatogram of VACSE showing the distribution of identified compounds. Each spectral peak corresponds to a specific phytochemical constituent, with the dominant peak representing the major compound in the extract.

The bioactive compounds of VACLE and VACSE were identified using GC–MS. The chromatograms are presented in Figure 1 and Figure 2, while retention time (RT), peak area (%), molecular formula (MF), and molecular weight (MW) are summarized in Table 2 and Table 3. A total of 55 compounds were detected in VACLE and 34 in VACS, indicating complex phytochemical profiles. VACLE was dominated by isoamyl formate (27.96%), 4H-pyran-4-one derivative (14.11%), and cyclohexanol derivative (14.05%), followed by Palmitic acid (9.32%), β-Monopalmitin (4.35%), and cis-vaccenic acid (3.72%). Minor constituents included anethole, carvacrol, eucalyptol, and phytol. VACSE showed a high abundance of fatty acids, mainly cis-Linoleic acid (40.58%) and Palmitic acid (21.87%), together accounting for more than 60% of the total composition. Additional compounds included p-Menthan-1-ol (10.26%), benzofuran derivatives (3.08%), and Octadecanoic acid (3.42%), as well as phytosterols such as β-sitosterol (2.12%) and γ-sitosterol. Overall, VACSE was enriched in fatty acids and sterols, whereas VACLE exhibited greater chemical diversity, particularly in esters and phenolic compounds, which may underlie differences in their biological activities.

### 3.3. TPC and TFC

The TPC of VACLE was 94.12 ± 3.65 mg GAE/g DW, while the TFC was 82.00 ± 2.15 mg QE/g DW. In comparison, VACSE exhibited lower TPC (54.12 ± 2.63 mg GAE/g DW) and TFC (42.00 ± 1.95 mg QE/g DW).

### 3.4. Antioxidant Activity of VACLE and VACSE

**Figure 3:**
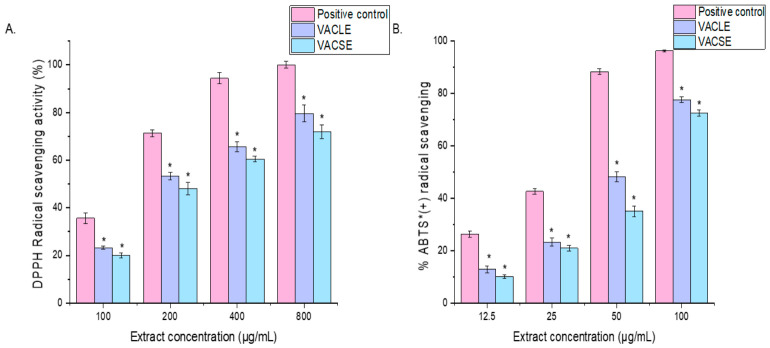
Antioxidant activity of VACLE and VACSE: (**A**) DPPH and (**B**) ABTS^+^ assays. Ascorbic acid served as the positive control. Data are mean ± SD (*n* = 3). * *p* < 0.05 vs. control. (+), radical cation.

Both extracts exhibited a concentration-dependent increase in antioxidant activity over the range of 100–800 µg/mL (Figure 3A,B) as determined by the DPPH assay. The ascorbic acid showed the strongest activity, reaching 99.99 ± 1.45% at 800 µg/mL, while VACLE and VACSE achieved 79.64 ± 3.61% and 64.93 ± 2.94%, respectively. At all tested concentrations, VACLE demonstrated higher activity than VACSE. The IC_50_ values further confirmed this trend, with the positive control showing the lowest IC_50_ (≈140 µg/mL), followed by VACLE (≈180 µg/mL) and VACSE (≈230 µg/mL), indicating lower antioxidant potency compared with the positive control.

A similar concentration-dependent pattern was observed in the ABTS^+^ assay (12.5–100 µg/mL). The positive control exhibited strong scavenging activity (96.36 ± 0.36% at 100 µg/mL), whereas VACLE and VACSE showed 77.64 ± 1.15% and 72.49 ± 1.19%, respectively. The IC_50_ values were calculated as ≈29.0 µg/mL for the positive control, ≈55 µg/mL for VACLE, and ≈70 µg/mL for VACSE. These findings indicate that VACLE exhibits stronger radical-scavenging activity than VACSE but remains less potent than the standard antioxidant. Overall, the lower IC_50_ values of VACLE compared with VACSE suggest a higher antioxidant potential, although both extracts were significantly less active than ascorbic acid (*p* < 0.05).

### 3.5. Antibacterial Effects of VACLE and VACSE

The antibacterial efficacy of VACLE and VACSE against six bacterial strains was assessed using agar well diffusion (Table 4 and Table 5). Both extracts showed dose-dependent inhibition, with significantly larger zones at 400–800 μg/mL (*p* < 0.05), albeit being smaller than those of chloramphenicol. VACLE showed greater potency (MIC: 6.25–50 μg/mL) than VACSE (12.5–100 μg/mL), with consistently wider inhibitory zones. Gram-positive bacteria, particularly *S. aureus*, were more susceptible, whereas *E. coli* and *P. aeruginosa* showed the weakest responses and highest MIC/MBC values.

### 3.6. Antifungal Activity

ZoI, MIC, and MFC were used to evaluate the antifungal activity of VACLE and VACSE against three *Candida* species (Table 6 and Table 7). Both extracts exhibited dose-dependent activity, with significantly lower ZoI than the positive control (fluconazole, 25 μg/mL; *p* < 0.05). VACSE showed greater antifungal potency, with *C. parapsilosis* being the most susceptible (MIC: 6.25 ± 2.26 μg/mL; MFC: 12.50 ± 2.67 μg/mL), followed by *C. glabrata. albicans* were the least sensitive. In contrast, VACLE exhibited weaker activity, with higher MIC and MFC values across all strains, although its strongest effect was observed against *C. parapsilosis*. Overall, VACSE demonstrated superior antifungal efficacy compared to VACLE.

### 3.7. Anticancer Activity of VACLE and VACSE

**Figure 4:**
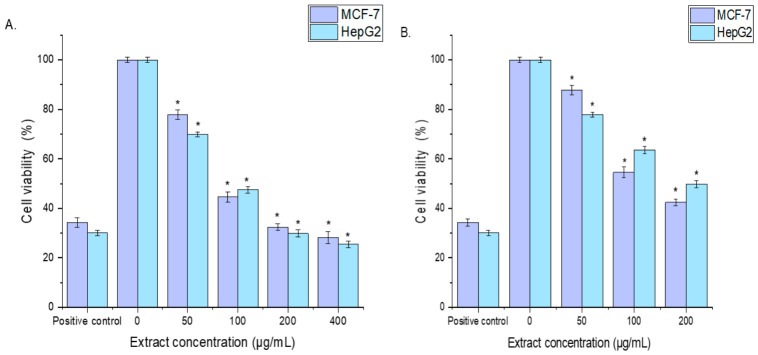
Effects of VACLE (**A**) and VACSE (**B**) on MCF-7 and HepG2 cell viability assessed by MTT assay following 24 h treatment (0–400 µg/mL). Values are mean ± SD (*n* = 3). * *p* < 0.05 versus positive control (cisplatin (30 µg/mL)).

The anticancer activity of VACLE and VACSE against MCF-7 and HepG2 cells was assessed using the MTT assay (Figure 4A,B). Both extracts exhibited concentration-dependent cytotoxic effects in the two cancer cell lines. However, VACLE demonstrated significantly greater potency than VACSE. At 400 µg/mL, VACLE reduced MCF-7 and HepG2 cell viability to 28.24 ± 2.45% and 25.43 ± 1.15%, respectively, whereas VACSE reduced viability to 38.24 ± 2.31% and 35.43 ± 1.25%. Consistent with these findings, the IC_50_ values of VACLE were markedly lower than those of VACSE, measuring 110.6 and 93.2 µg/mL against MCF-7 and HepG2 cells, respectively, compared with 193.8 and 247.5 µg/mL for VACSE. Overall, HepG2 cells were more sensitive to VACLE treatment, indicating that VACLE possesses stronger anticancer activity than VACSE under the tested conditions.

### 3.8. Effect of VACLE and VACSE on Apoptosis-Related Markers

**Figure 5:**
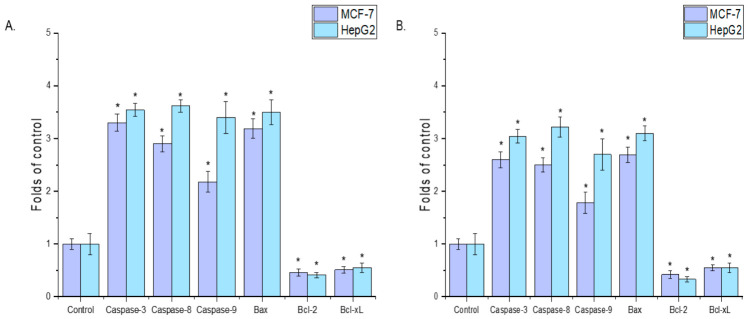
Effects of VACLE (**A**) and VACSE (**B**) on induction of apoptosis in human MCF-7 and HepG2 cells. The cells were treated without or with IC_50_ values of VACLE and VACSE for 48 h, collected RNA, isolated, and subjected to rRT-PCR. VACLE and VACSE significantly stimulate the activity of a subset of apoptotic genes (*caspase-3*, -*8*, -*9*, and *Bax*) and anti-apoptotic genes (*Bcl-xL* and *Bcl-2*) compared to control cells. The results are presented as the mean ± SD from three independent experiments (* *p* < 0.05 compared to untreated cells (control)).

Exposure of MCF-7 and HepG2 cells to VACLE and VACSE significantly regulated apoptosis-associated genes (Figure 5A,B). VACLE treatment was associated with the strongest transcriptional response, with upregulation of caspase-3, caspase-8, caspase-9, and Bax, and downregulation of Bcl-2 and Bcl-xL at the mRNA level. These expression-level changes are consistent with engagement of intrinsic and extrinsic apoptotic pathways but require protein-level confirmation (Western blot, caspase activity assay, or flow cytometric Annexin V staining), which was beyond the scope of the present screening study. VACSE also altered the expression of these genes but with lower fold changes. Collectively, the data demonstrate that VACLE exerts a more pronounced effect on apoptosis-related gene expression than VACSE.

### 3.9. Antidiabetic Activities

#### α-Amylase and α-Glucosidase Inhibitory Activities

**Figure 6:**
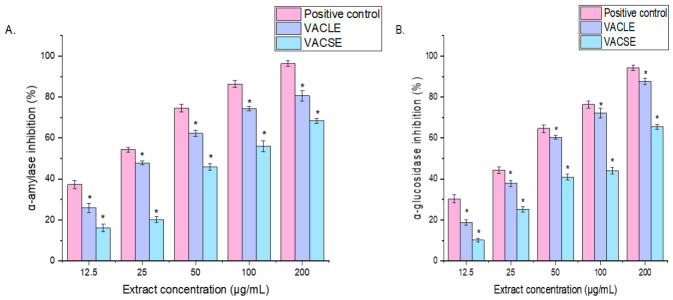
Inhibitory effects of VACLE and VACSE on (**A**) α-amylase and (**B**) α-glucosidase activities at concentrations ranging from 12.5 to 200 µg/mL. Values are expressed as mean ± SD (*n* = 3). * *p* < 0.05 versus positive control (acarbose).

The antidiabetic activities of VACLE and VACSE were evaluated for their inhibitory effects on α-amylase and α-glucosidase. Both extracts exhibited concentration-dependent inhibition over the tested range (12.5–200 µg/mL). VACLE demonstrated stronger inhibitory activity than VACSE, with lower IC_50_ values for α-amylase (28.7 vs. 70.1 µg/mL) and α-glucosidase (38.6 vs. 127.8 µg/mL). However, both extracts were less potent than the positive control, acarbose (21.85 and 32.0 µg/mL, respectively). Overall, VACLE showed superior inhibitory potential against both enzymes, as illustrated in Figure 6A,B.

## 4. Discussion

The chemical profiles of VACLE and VACSE differed significantly. The most noticeable difference between the two samples is the presence of isoamyl formate (27.96%) in the leaf extract, but its absence in the seed extract. Such a compound needs careful interpretation, as its presence in extremely large quantities may reflect its formation during extraction (e.g., esterification during methanolic extraction), which warrants future investigation with another extraction solvent, such as hexane. While VACLE had a significant amount of the 4H-pyran-4-one derivative, VACSE was rich in cis-linoleic and Palmitic acids, which were less plentiful in VACLE. The presence of substantial quantities of unsaturated fatty acids in both extracts suggests that these substances may have anti-inflammatory or immunomodulatory properties. However, this is still a hypothesis. Direct assessment of isolated fatty acids or fractionated extracts is required to determine their individual and combined contributions to the observed bioactivities. This pattern indicates differential production in leaf and fruit tissues, consistent with prior results showing that Vitex fruits contain more fatty acids and diterpenes and exhibit a greater buildup of volatile terpenoids in leaves. Phytol, neophytadiene, Squalene, β-sitosterol, and other diterpenoid derivatives detected in both samples have pharmacological implications. According to recent research, the diterpenes in *V. agnus-castus* fruits act as dopaminergic D2 receptor agonists, inhibiting prolactin release and alleviating premenstrual syndrome symptoms, suggesting that triterpenes play a significant role in mediating dopaminergic activities previously attributed solely to diterpenes [5]. These compounds are known for their anti-inflammatory and cholesterol-lowering actions rather than direct radical scavenging [35,36], in addition to their detergent-like effect on bacterial cell integrity [37]. Furthermore, previous studies have reported that free fatty acids can act through various antifungal mechanisms, including alterations in membrane integrity, inhibition of hyphal development, and impairment of ergosterol production [38,39]. Also, phytol, squalene, neophytadiene, and villosin, all of which have previously been reported to have anticancer effects [40,41]. The spatial arrangement and substitutions inside the side chain determine dopamine action, according to structural studies. However, it is vital to emphasize that a connection between metabolite presence and observed bioactivity does not imply causality. The antibacterial activity of these fatty acids in molecular docking investigations of allied *Vitex* species [42]. suggests a plausible hypothesis, but direct confirmation would necessitate fractionation studies or isolated compound testing. The current data support this idea, but do not prove causality.

Recently, the effect of VACLE collected in Algeria on the DNA gyrase enzymes of *E. coli*, *S. aureus*, and *B. subtilis* was investigated using molecular docking, with linolenic and palmitic acids showing significant interactions with all three bacterial types, indicating antibacterial potential [42]. The ethyl acetate extract showed antioxidant activity (IC_50_ = 15.68 ± 1.51 μg/mL in DPPH assay), while the dichloromethane fraction had mild butyrylcholinesterase inhibitory activity (IC_50_ = 133.54 ± 1.45 μg/mL) [43]. Both properties were compatible with the extracts’ fatty acid content. This implies that Pyranone derivatives and certain diterpenes, rather than nitrogenous chemicals, may be the key contributors to chemopreventive efficacy. Comparative investigations of *V. rotundifolia* and *V. trifolia* reveal shared diterpenoid profiles, with rotundifuran as a common metabolite [44,45].

VACLE had significantly higher TPC and TFC compared to VACSE. Our TPC and TFC values are consistent with previous reports that VACLE contains more phenolics than VACSE [46,47]. The high TPC and TFC in VACLE align well with known values for other medicinal plants in the *Lamiaceae* family, such as *Salvia officinalis* [48]. A previous study showed that casticin, a flavonoid compound isolated from *V. agnus-castus* fruit, has strong lipid peroxidation inhibition activity, surpassing that of ascorbic acid (IC_50_ = 0.703 mM) [49]. This specificity in flavonoid and non-flavonoid phenolic accumulation justifies the traditional differential use of these plant parts, as leaves may be more effective for treating diseases associated with oxidative stress, whereas seeds are useful due to their high levels of fatty acids and sterols [50].

The antioxidant activities of VACLE and VACSE were investigated, revealing a concentration-dependent reduction in capacity and free radical scavenging ability, with VACLE showing stronger activity than VACSE. This pattern paralleled VACLE’s higher TPC and TFC values compared to VACSE, and is consistent with the well-documented antioxidant contribution of flavonoid-rich extracts [49]; however, direct causal attribution requires bioactivity-guided fractionation. This could be attributable to VACLE’s higher TPC and TFC values of VACLE compared to VACSE. VACLE’s increased antioxidant activity is mostly due to higher flavonoid concentrations [49]. In agreement with our findings, a 2025 investigation by Bramki et al. reported that the ethyl acetate fraction of *V. agnus-castus* leaves demonstrated considerable DPPH radical-scavenging activity (IC_50_ = 15.68 ± 1.51 µg/mL) and mild butyrylcholinesterase inhibition (IC_50_ = 133.54 ± 1.45 µg/mL) [43]. In their 2023 investigation, Boujbiha and his colleagues identified *V. agnus-castus* leaves with a high TPC (78.53 ± 2.08 mg GAE/g dried extract) and antioxidant activity (IC_50_ = 0.64 mg/mL) [50]. Nevertheless, IC_50_ comparisons between *V. agnus-castus* samples should be approached with caution due to factors such as differing extraction processes, plant origins, and test conditions. In comparison to other *Lamiaceae*, VACLE exhibits moderate antioxidant activity, with the hydroethanolic extracts of *Origanum compactum* (radical-scavenging activity of 470.90 mg EAA/g E) [51], and the essential oil of *S. officinalis* (IC_50_ = 6.16 mg/mL) [52]. VACLE’s increased antioxidant activity corresponds with higher TPC and TFC levels. This link is compatible with the known radical-scavenging characteristics of phenolic chemicals, although it does not prove causation. Other compound classes (e.g., terpenoids, fatty acids) or synergistic interactions among numerous ingredients could account for the reported effectiveness. Future fractionation experiments are required to identify the particular chemicals involved.

The antibacterial testing results show that VACLE is more effective than VACSE, with MICs ranging from 6.25 to 50 μg/mL for VACLE and 12.5 to 100 μg/mL for VACSE. Overall, both extracts inhibited all tested strains in a dose-dependent manner. Gram-positive bacteria, such as *S. aureus*, were found to be more susceptible than Gram-negative bacteria. This is consistent with the principle of natural Gram-negative resistance, which arises from lipopolysaccharides in the outer membrane that prevent hydrophobic substances from entering [53]. In alignment with previous studies, the Algerian leaves of *V. agnus-castus* showed significant antibacterial effects, with molecular docking analysis indicating interactions of linolenic acid, palmitic acid, and phytol with the DNA gyrase of *E. coli*, *S. aureus*, and *B. subtilis* [43]. Also, Zhelev et al. (2022) reported the antibacterial properties of Bulgarian extracts from *V. agnus-castus* against the Gram-positive bacteria *S. aureus*, *B. subtilis*, and *Kocuria rhizophila*; the Gram-negative bacterium *Salmonella Abony;* and the yeast *Saccharomyces cerevisiae* [54]. Furthermore, essential oils of *Origanum compactum* and *Thymus zygis* (*Lamiaceae)* exhibited effective antibacterial activity consistent with their thymol and carvacrol content [55], whereas *S. officinalis* showed activity similar to VACLE against *S. aureus* and *E. coli* [56].

The antifungal test demonstrated clear organ specificity. While VACLE was more potent in antibacterial testing than VACSE, the seed extract performed significantly better in antifungal tests. This discrepancy in data suggests that the underlying mechanisms of action in antibacterial and antifungal trials differ considerably. *C. parapsilosis*, the *Candida* species most susceptible to the examined materials, has higher membrane fluidity and susceptibility to fatty acids when compared to *C. albicans* [57], as evidenced by variations in cell wall composition and efflux pump expression [58]. The low MFC/MIC ratio of VACSE against *C. parapsilosis* indicates its fungicidal activity. In general, the results confirm prior findings for the *Vitex* and *Lamiaceae* genera, such as antifungal activity of *V. negundo* seeds against *C. albicans* (29.73% inhibition) [59], *O. vulgare* essential oil against *C. albicans* (MIC% 0.25–1%), *C. dubliniensis* (MIC% 0.25–1%), *C. parapsilosis* (MIC% 0.25–1%), *C. lusitanie* (MIC% 0.12–1%), and *C. Krusei* (MIC% 0.25–1%) [60], and *S. officinalis* against *C. albicans* (2780 μg/mL) [61].

The anticancer screening results demonstrated that VACLE possesses substantially higher cytotoxic activity than VACSE against both MCF-7 and HepG2 cell lines. Among the tested cell lines, HepG2 cells were more responsive to VACLE treatment, exhibiting a lower IC_50_ value (93.2 µg/mL) than MCF-7 cells (110.6 µg/mL), compared with 193.8 and 247.5 µg/mL for VACSE. This differential sensitivity may reflect variations in cellular metabolism, molecular targets, or uptake of the bioactive compounds present in the leaf extract. The effectiveness of the VACLE is corroborated by Bramki et al. (2025), who reported cytotoxic effects of VACLE on cancer cells via molecular docking of linoleic acid and palmitic acid with the DNA gyrase enzyme [43]. Other studies reported the cytotoxic activities of *V. agnus-castus* seeds against HGC27 (IC_50_: 26.6 μg/mL), MKN45 (IC_50_: 33 μg/mL), and AGS (IC_50_: 31.4 μg/mL) gastric cancer cell lines [10], and MCF-7 breast cancer cells, resulting in DNA damage and triggering apoptosis [62]. Furthermore, both extracts induced apoptosis by activating the caspase pathway and regulating *Bcl-2* family gene levels. In this regard, upregulation of *caspase-3* (3.3–3.54-fold), *caspase-8* (2.9–3.62-fold), *caspase-9* (2.18–3.40-fold), and *Bax* (3.19–3.50-fold) in VACLE-treated cells, accompanied by downregulation of Bcl-2 and Bcl-xL to 0.41–0.46 and 0.51–0.55-fold, respectively, indicates the activation of both the intrinsic and extrinsic apoptotic pathways. Similarly, treatment with the seed extract enhanced expression of *caspase-3* (2.6–3.04-fold), *caspase-8* (2.5–3.22-fold), *caspase-9* (1.78–2.70-fold), and *Bax* (2.69–3.10-fold), while decreasing *Bcl-2* to 0.33–0.42-fold. Consistent with our findings, a previous study reported that an aqueous VACSE reduced the viability of gastric cancer cells by activating caspase-3, reducing Bcl-2 expression, and inducing G0/G1-phase cell-cycle arrest [10]. Another study found that a methanolic VACLE effectively inhibited HepG2 cells (IC_50_ = 17.61 ± 0.15 mg/mL) by increasing p53 protein levels and altering the Bax/Bcl-2 ratio [63]. Extracts from other plants in the *Lamiaceae* family had similar anticancer effects, such as *S. officinalis* and *Rosmarinus officinalis* against MCF-7 (breast), A594 (lung), and Lovo (colorectal) [64], and *Origanum* spp. against CaCo-2, MCF-7, and A549 [65]. VACLE’s moderate potency, paired with its favorable mechanism of action (dual-pathway caspase activation), makes this extract a good candidate for further fractionation and bioactivity-guided isolation of lead anticancer drugs. The observed upregulation of caspase-3, caspase-8, caspase-9, and Bax mRNAs, accompanied by downregulation of Bcl-2 and Bcl-xL mRNAs, is consistent with the hypothesis that VACLE and VACSE may induce apoptosis via both intrinsic and extrinsic pathways. However, the present data are limited to transcript-level measurements. Without corresponding protein-level quantification, direct caspase activity assays, or functional apoptosis readouts (e.g., Annexin V staining, TUNEL, or flow cytometric assessment of sub-G1 populations), the mechanistic interpretation remains speculative. These findings should therefore be considered hypothesis-generating and in need of confirmatory studies.

In the antidiabetic experiment, VACLE inhibited α-amylase and α-glucosidase more effectively than VACSE, and both samples were less effective than acarbose. As mentioned before, the high TPC and TFC values might contribute to antidiabetic activities, as flavonoids function as competitive inhibitors of carbohydrate hydrolases by interacting with enzyme active sites [66]. These results are consistent with previous investigations of the genus *Vitex* [67]. A previous study reported that the hydroalcoholic extract of *V. agnus-castus* fruit reduced serum glucose and D-galactose levels in diabetic mice, accompanied by an increase in the Insulin level [11]. Berrani et al. (2018) reported that oral administration of 300 mg/kg of the methanolic extract of *V. agnus-castus* at 300 mg/kg induced a short-term decrease in blood glucose and improved the lipid profile in a diabetic mouse model [12]. Similarly, other *Lamiaceae* family members showed potent antidiabetic activity, such as *V. negundo* leaf extract [68], *O. compactum*, *O. elongatum*, and *O. vulgare*, which demonstrated α-amylase and α-glucosidase inhibitory activity [69,70]. Overall, both extracts exhibited inhibitory activity against α-amylase and α-glucosidase enzymes, with VACLE showing greater potency than VACSE. However, these findings are based solely on *in vitro* enzyme inhibition assays and should be considered preliminary. Further studies using cellular and *in vivo* models are required to confirm the antidiabetic potential and underlying mechanisms of the extracts.

VACLE exhibited higher total phenolic and flavonoid contents than VACSE and generally demonstrated stronger antioxidant, antibacterial, anticancer, and antidiabetic activities. Although these findings are consistent with the well-established contribution of phenolic and flavonoid compounds to biological activity, the present study does not establish a direct causal relationship between total phenolic/flavonoid levels and the observed bioactivities. The measured TPC and TFC values provide only an estimate of overall compound abundance and do not identify the specific constituents responsible for the biological effects. Therefore, the observed associations should be considered correlative rather than mechanistic.

The present study provides one of the first integrated comparisons of VACLE and VACSE, combining GC-MS-based metabolomics with multidimensional bioactivity profiling. In this discussion, we distinguish three categories of interpretation: directly demonstrated findings—those supported by experimental data presented in this study (e.g., IC_50_ values, MIC determinations, gene expression changes); literature-supported hypotheses—plausible explanations consistent with published studies on *Vitex* species or related plants, which require direct validation in future work; and speculative interpretations—tentative. This distinction is meant to promote scientific transparency and help guide future research. Major strengths of the present study are the unique phytochemical composition of *V. agnus-castus* leaves, indicating specific bioactivity of tissues, as evidenced by better antioxidant, antibacterial, anticancer, and antidiabetic properties of the leaves and comparatively more antifungal activity of the seeds; and elucidation of the apoptotic mechanism through the activation of caspase-8 and caspase-9 signaling pathways.

However, several limitations should be acknowledged. The putative nature of metabolite identifications derived from the GC–MS data cannot be ignored, as standards were available for most of the detected compounds. The RI validation procedure was carried out only for some of the metabolites. The aforementioned issue is especially relevant in cases involving closely related molecules such as terpenes and fatty acids. In future research efforts, the use of complementary analytical approaches (for instance, liquid chromatography–tandem mass spectrometry (LC–MS/MS) with authentic standards) is recommended. It should also be noted that the phytochemical composition was evaluated based on GC–MS peak area normalization. Therefore, the reported values represent relative abundances and may not accurately reflect the absolute concentrations of individual compounds because of differences in ionization efficiency and detector response. An additional methodological limitation is the absence of multivariate statistical integration (e.g., partial least squares regression, or correlation heatmaps) linking metabolite composition with biological activity profiles. Such analyses would help identify which metabolites or metabolite classes are most strongly associated with specific bioactivities and would test whether the observed organ-specific differences are statistically significant in multivariate space. We acknowledge that the current study remains descriptive in this regard. Future investigations should include these analytical approaches to move beyond descriptive comparison toward predictive modeling of tissue-specific bioactivity. Another limitation is that all biological evaluations were performed *in vitro*; therefore, the results cannot be directly extrapolated to *in vivo* conditions, where bioavailability, metabolism, and pharmacokinetic factors may influence biological activity. In addition, cytotoxicity was assessed only in cancer cell lines, preventing evaluation of selectivity toward malignant cells and limiting safety assessment. Future studies should include normal human cell lines and *in vivo* models to establish selectivity, safety, and therapeutic potential. Also, they do not consider Absorption, Distribution, Metabolism, and Excretion (ADME) processes that occur during *in vivo* studies. One limitation of the present study is that antioxidant activity was evaluated exclusively using chemical assays (DPPH and ABTS^+^). Although these methods are widely used for preliminary screening of radical-scavenging activity, they do not fully represent the complexity of antioxidant mechanisms in biological systems. Another limitation is that the postulated apoptotic pathway was based only on gene expression data. Transcript abundance is not always associated with protein activity, post-translational changes, or functional effects. Furthermore, we did not quantify ROS, evaluate mitochondrial membrane potential, or confirm apoptosis using flow cytometry. Therefore, future studies should include cell-based oxidative stress models and *in vivo* experiments to confirm the biological antioxidant potential and physiological relevance of the extracts. Another limitation of the present study is that the proposed apoptotic mechanism was inferred solely from gene expression data. Further protein-level and functional studies are required to validate the underlying molecular pathways. Finally, the study did not analyze the potential synergistic or antagonistic interactions of the investigated substances, which could be significant given the phytochemical complexity. Future research should prioritize the aforementioned approaches to address the limitations identified in this study. Another aspect to consider in the conduct of pharmacokinetic tests is determining bioavailability, metabolism, and distribution. Lastly, due to the high abundance of isoamyl formate in VACLE, this compound needs to be verified independently by different extraction methodologies.

## 5. Conclusions

VACLE outperformed VACSE *in vitro* in terms of antioxidative, antibacterial, anticancer, and antidiabetic activity. These findings indicate the possibility for tissue-specific uses, but do not show therapeutic efficacy. VACLE generally showed stronger antioxidant, antibacterial, anticancer, and enzyme inhibitory activities, whereas VACSE displayed greater antifungal activity. VACLE and VACSE both elicited transcriptional changes consistent with caspase-mediated apoptosis at the mRNA level, with VACLE producing the stronger effect; protein-level validation is required for mechanistic confirmation. Future research should focus on bioactivity-guided fractionation to establish the chemical composition linked with biological activity. This is especially important for identifying isoamyl formate and the 4H-pyran-4-one derivative in leaves; further validation in animal models; elucidating molecular mechanisms through transcriptomic or proteomic studies; toxicity testing in human cells to calculate therapeutic indices; developing formulations to improve bioavailability; and conducting clinical trials.

## Figures and Tables

**Table 1:** The primer sequences of various genes involved in apoptosis and anti-apoptotic genes.

Gene Name	Primer’s Sequence	Reference
*Caspase-3*	F: 5′-GCTGGATGCCGTCTAGAGTC-3′	[29]
	R: 5′-ATGTGTGGATGATGCTGCCA-3′	
*Caspase-8*	F: 5′-AGAAGAGGGTCATCCTGGGAGA-3′	[30]
	R: 5′-TCAGGACTTCCTTCAAGGCTGC-3′	
*Caspase-9*	F: 5′-ATTGCACAGCACGTTCACAC-3′	[29]
	R: 5′-TATCCCATCCCAGGAAGGCA-3′	
*Bax*	F: 5′-GAGCTAGGGTCAGAGGGTCA-3′	[29]
	R: 5′-CCCCGATTCATCTACCCTGC-3′	
*Bcl-2*	F: 5′-ACCTACCCAGCCTCCGTTAT-3′	[29]
	R: 5′-GAACTGGGGGAGGATTGTGG-3′	
*Bcl-XL*	F: 5′-CAGAGCTTTGAACAGGTAG-3′	[31]
	R: 5′-GCTCTCGGGTGCTGTATTG-3′	
	R: 5′-GGGCGGATTAGGGCTTCC-3′	
*GAPDH*	F: 5′-CGGAGTCAACGGATTTGGTC-3′	[32]
	R: 5′-AGCCTTCTCCATGGTCGTGA-3′	

**Table 2:** GC-MS compounds in VACLE.

No.	RT	Area	Area%	Name	MF	MW	CAS Number	IUPAC Name	Class	Confidence Level
1	9.798	4,399,318	0.25	2,5-Dimethylfuran-3,4(2H,5H)-dione	C_6_H_8_O_3_	128	68755-49-7	2,5-dimethylfuran-3,4(2H,5H)-dione	Furanones (Flavor)	Level 3
2	10.122	6,661,994	0.38	Mequinol	C_7_H_8_O_2_	124	150-76-5	4-methoxyphenol	Methoxyphenols	Level 3
3	12.058	243,135,780	14.11	4H-Pyran-4-one, 2,3-dihydro-3,5-dihydroxy-6-methyl-	C_6_H_8_O_4_	144	28564-83-2	3,5-dihydroxy-6-methyl-2,3-dihydropyran-4-one	Pyranone derivative	Level 3
4	12.717	242,106,406	14.05	Cyclohexanol, 1-methyl-4-(1-methylethyl)-	C_10_H_20_O	165	21129-27-1	1-methyl-4-(propan-2-yl)cyclohexan-1-ol	Monoterpenoids	Level 2
5	13.47	482,192,147	27.96	Isoamyl formate	C_6_H_12_O_2_	116	110-45-2	3-methylbutyl formate	Carboxylic acid derivatives	Level 2
6	14.709	8,937,825	0.518	Benzofuran, 2,3-dihydro-	C_8_H_8_O	120	496-16-2	2,3-dihydro-1-benzofuran	Coumaran	Level 3
7	14.963	9,295,676	0.539	Anisaldehyde	C_8_H_8_O_2_	136	123-11-5	4-methoxybenzaldehyde	Benzoyl derivatives	Level 3
8	15.111	9,650,458	0.55	5-Hydroxymethylfurfural	C_6_H_6_O_3_	126	67-47-0	5-(hydroxymethyl)furan-2-carbaldehyde	Bicyclic ether (1,8-Cineole)	Level 2
9	15.729	11,345,290	0.65	Anethole	C_10_H_12_O	148	104-46-1	1-methoxy-4-[(E)-prop-1-enyl]benzene	Diterpene hydrocarbon	Level 2
10	16.232	2,572,093	0.14	Phenol, 2-methyl-5-(1-methylethyl)-	C_10_H_14_O	150	499-75-2	2-methyl-5-(propan-2-yl)phenol	Diterpene lactone	Level 3
11	16.462	2,204,623	0.12	Carvacrol	C_10_H_14_O	150	499-75-2	2-methyl-5-(propan-2-yl)phenol	Diterpene lactone	Level 2
12	16.536	6,237,580	0.36	Benzeneacetic acid, 4-hydroxy-	C_8_H_8_O_3_	152	156-38-7	2-(4-hydroxyphenyl)acetic acid	Diterpenoid (Chlorophyll derivative)	Level 3
13	17.535	5,474,116	0.32	Eucalyptol	C_10_H_18_O	154	470-82-6	1,3,3-trimethyl-2-oxabicyclo [2.2.2]octane	Diterpenoid (Phytol derivative)	Level 2
14	18.604	1,331,248	0.07	1-(3,6,6-Trimethyl-1,6,7,7a-tetrahydrocyclopenta[c]pyran-1-yl)ethanone	C_13_H_18_O_2_	206	Not Found	1-(3,6,6-Trimethyl-1,6,7,7a-tetrahydrocyclopenta[c]pyran-1-yl)ethanone	Diterpenoid (Phytol isomer)	Level 4
15	18.804	2,327,627	0.13	2,11-Dioxabicyclo [4.4.1]undeca-3,5-dien-10-one, 1,3,7,7-tetramethyl-	C_13_H_18_O_3_	222	70412-52-1	1,3,7,7-tetramethyl-2,11-dioxabicyclo [4.4.1]undeca-3,5-dien-10-one	Dioxepines	Level 3
16	20.686	3,310,259	0.19	4-(2,6,6-Trimethylcyclohexa-1,3-dienyl)but-3-en-2-one	C1_3_H_18_O	190	1203-08-3	(E)-4-(2,6,6-trimethylcyclohexa-1,3-dien-1-yl)but-3-en-2-one	Sesquiterpenoids	Level 3
17	22.943	12,230,962	0.71	3′,5′-Dimethoxyacetophenone	C_10_H_12_O_3_	180	39151-19-4	1-(3,5-dimethoxyphenyl)ethan-1-one	Ester, flavor compound	Level 3
18	23.209	7,389,682	0.42	1-Oxaspiro [2.5]octane, 5,5-dimethyl-4-(3-methyl-1,3-butadienyl)-	C_14_H_22_O	206	Not Found	5,5-dimethyl-4-[(1E)-3-methylbuta-1,3-dienyl]-1-oxaspiro [2.5]octane	Fatty alcohol	Level 4
19	23.471	9,107,663	0.52	p-Cymene-2,5-diol	C_10_H_14_O_2_	166	2217-60-9	2-methyl-5-(propan-2-yl)benzene-1,4-diol	Fatty alcohol acetate (Pheromone)	Level 3
20	23.546	14,657,809	0.8	1-(4-Methoxyphenyl)propane-1,2-diol	C_10_H_14_O_3_	182	51410-48-1	1-(4-methoxyphenyl)propane-1,2-diol	Phenylpropanes	Level 3
21	24.267	2,270,146	0.13	Megastigmatrienone A	C_13_H_18_O	190	38818-55-2	(4Z)-4-[(E)-but-2-enylidene]-3,5,5-trimethylcyclohex-2-en-1-one	Furanone (flavor)	Level 3
22	24.938	2,293,192	0.14	Butan-2-one, 4-(3-hydroxy-2-methoxyphenyl)-	C_11_H_14_O_3_	194	303187-89-5	4-(3-hydroxy-2-methoxyphenyl)butan-2-one	Long-chain monounsaturated fatty acid (Methoxyphenols)	Level 3
23	27.075	2,884,427	0.16	Trans-Coniferyl alcohol	C_10_H_12_O_3_	180	458-35-5	(E)-4-(3-hydroxyprop-1-en-1-yl)-2-methoxyphenol	Long-chain monounsaturated fatty acid (Methoxyphenols)	Level 3
24	27.317	15,636,768	0.91	Tetradecanoic acid	C_14_H_28_O_2_	228	544-63-8	tetradecanoic acid	Fatty Acyls	Level 2
25	27.716	15,987,734	0.92	Pentadecanoic acid	C_15_H_30_O_2_	242	1002-84-2	pentadecanoic acid	Fatty Acyls	Level 2
26	28.354	16,183,773	0.93	2-Methyl-4-(2,6,6-trimethylcyclohex-1-enyl)but-2-en-1-ol	C_14_H_24_O	208	53892-68-5	(E)-2-methyl-4-(2,6,6-trimethylcyclohexen-1-yl)but-2-en-1-ol	Monoterpenoids	Level 3
27	28.806	2,799,197	0.16	Neophytadiene	C_20_H_38_	278	504-96-1	7,11,15-trimethyl-3-methylidenehexadec-1-ene	Diterpenoids	Level 2
28	28.917	11,752,161	0.68	2-Pentadecanone, 6,10,14-trimethyl-	C_18_H_36_O	268	502-69-2	6,10,14-trimethylpentadecan-2-one	Sesquiterpenoids	Level 3
29	29.055	10,475,669	0.61	1b,5,5,6a-Tetramethyl-octahydro-1-oxa-cyclopropa[a]inden-6-one	C_13_H_20_O_2_	208	Not Found	1b,5,5,6a-tetramethyl-2,3,4,5a-tetrahydro-1aH-indeno [1,2-b]oxiren-6-one	Norisoprenoid	Level 4
30	29.21	4,024,844	0.23	4-(1,5-Dihydroxy-2,6,6-trimethylcyclohex-2-enyl)but-3-en-2-one	C_13_H_20_O_3_	224	Not Found	4-[(2S,4S)-2,4-dihydroxy-2,6,6-trimethylcyclohexylidene]but-3-en-2-one	Fatty Acyls	Level 4
31	29.425	6,908,994	0.41	3-Hydroxy-5,6-epoxy-β-ionone	C_14_H_22_O_2_	222	172705-14-5	2,2,6-trimethyl-1-[(1E)-3-methylbuta-1,3-dienyl]-7-oxabicyclo [4.1.0]heptan-3-ol	Oxepanes	Level 3
32	29.805	4,108,453	0.23	3,7,11,15-Tetramethyl-2-hexadecen-1-ol	C_20_H_40_O	296	102608-53-7	(E)-3,7,11,15-tetramethylhexadec-2-en-1-ol	Diterpenoids	Level 3
33	30.835	1,608,401	0.09	Picrotoxinin	C1_5_H_16_O_6_	292	17617-45-7	(1R,3R,5S,8S,9R,12S,13R,14R)-1-hydroxy-13-methyl-14-prop-1-en-2-yl-4,7,10-trioxapentacyclo [6.4.1.19,12.03,5.05,13]tetradecane-6,11-dione	Phenolic monoterpenoid	Level 3
34	31.361	1,786,746	0.1	17-Octadecynoic acid	C_18_H_32_O_2_	280	34450-18-5	octadec-17-ynoic acid	Phenolic monoterpenoid	Level 3
35	32.03	160,702,963	9.32	Palmitic acid	C_16_H_32_O_2_	256	57-10-3	hexadecanoic acid	Fatty Acyls	Level 2
36	32.518	9,989,076	0.57	trans-2-Hexadecenoic acid	C_16_H_30_O_2_	254	929-79-3	(E)-hexadec-2-enoic acid	Fatty Acyls (Flavors)	Level 3
37	33.586	4,057,579	0.23	12-Methyl-E,E-2,13-octadecadien-1-ol	C_19_H_36_O	280	874197-21-4	(2E,13E)-12-methyloctadeca-2,13-dien-1-ol	Fatty Acyls	Level 3
38	33.743	4,715,464	0.27	Margaric acid	C_17_H_34_O_2_	270	506-12-7	heptadecanoic acid	Fatty Acyls	Level 2
39	34.377	9,672,006	0.56	Phytol	C_20_H_40_O	296	150-86-7	(E,7R,11R)-3,7,11,15-tetramethylhexadec-2-en-1-ol	Diterpenoids	Level 2
40	35.088	19,106,369	1.11	cis-Linoleic acid	C_18_H_32_O_2_	280	60-33-3	(9Z,12Z)-octadeca-9,12-dienoic acid	Fatty Acyls	Level 2
41	35.248	64,221,102	3.72	cis-Vaccenic acid	C_18_H_34_O_2_	282	506-17-2	(Z)-octadec-11-enoic acid	Fatty Acyls	Level 2
42	35.706	39,838,818	2.31	Octadecanoic acid	C_18_H_36_O_2_	284	57-11-4	octadecanoic acid	Fatty Acyls	Level 2
43	36.042	8,423,453	0.48	Linoelaidic acid	C_18_H_32_O_2_	280	506-21-8	(9E,12E)-octadeca-9,12-dienoic acid	Fatty Acyls	Level 2
44	36.777	3,950,509	0.22	Z,Z-10,12-Hexadecadien-1-ol acetate	C_18_H_32_O_2_	280	73829-33-1	(10Z,12Z)-hexadeca-10,12-dien-1-yl acetate	Sesquiterpenoid	Level 3
45	37.16	1,631,096	0.09	3-Hydroxy-α-ionene	C_13_H_20_O_2_	208	Not Found	3-hydroxy-alpha-ionone	Sesquiterpenoid	Level 4
46	37.336	2,678,685	0.15	Oleic Acid	C_18_H_34_O_2_	282	112-80-1	(Z)-octadec-9-enoic acid	Sesquiterpenoid	Level 2
47	37.498	2,678,418	0.15	4,8,12,16-Tetramethylheptadecan-4-olide	C_21_H_40_O_2_	324	96168-15-9	5-methyl-5-(4,8,12-trimethyltridecyl)oxolan-2-one	Terpene lactone	Level 3
48	37.703	2,921,771	0.16	Villosin	C_20_H_28_O_2_	300	160598-92-5	4-[(E)-2-[(1S,4aS,8aS)-5,5,8a-trimethyl-2-methylidene-3,4,4a,6,7,8-hexahydro-1H-naphthalen-1-yl]ethenyl]-2H-furan-5-one	Terpene lactone	Level 3
49	38.599	2,617,246	0.15	Hexanoic acid, 4-hexadecyl ester	C_22_H_44_O_2_	340	14331-11-4	hexadecyl hexanoate	Norisoprenoid	Level 3
50	39.403	1,937,535	0.11	cis-10-Nonadecenoic acid	C_19_H_36_O_2_	296	73033-09-7	(Z)-nonadec-10-enoic acid	Sesquiterpene	Level 3
51	41.54	75,164,138	4.35	β-Monopalmitin	C_19_H_38_O_4_	330	23470-00-0	2-hydroxy-1-(hydroxymethyl)ethyl hexadecanoate	Terpenoid, alcohol	Level 2
52	45.064	22,047,193	1.27	α-Monostearin	C_21_H_42_O_4_	356	123-94-4	2,3-dihydroxypropyl octadecanoate	Glycerolipids	Level 2
53	46.045	4,928,297	0.28	cis-13-Eicosenoic acid	C_20_H_38_O_2_	310	17735-94-3	(Z)-icos-13-enoic acid	Triterpene hydrocarbon	Level 3
54	47.96	13,240,380	0.76	Squalene	C_30_H_50_	410	111-02-4	(6E,10E,14E,18E)-2,6,10,15,19,23-hexamethyltetracosa-2,6,10,14,18,22-hexaene	Triterpenoids	Level 2
55	48.726	6,393,446	0.37	Epoxyoleic acid	C_18_H_34_O_3_	298	24560-98-3	cis-8-(3-octyloxiran-2-yl)octanoic acid	Fatty Acyls	Level 3

Compound identifications are hypothetical; confidence levels (Level 2, 3, or 4 according to MSI norms) are supplied in the ‘Confidence level’ column.

**Table 3:** GC-MS compounds in VACSE.

Peak	RT	Area	Area%	Name	MF	MW	CAS Number	IUPAC Name	Class	Confidence Level
1	12.324	263,431,988	10.26	p-Menthan-1-ol	C_10_H_20_O	165	21129-27-1	1-methyl-4-(propan-2-yl)cyclohexan-1-ol	Monoterpenoid	Level 2
2	12.733	5,912,740	0.23	Menthol	C_10_H_20_O	156	2216-51-5	(1R,2S,5R)-5-methyl-2-propan-2-ylcyclohexan-1-ol	Monoterpenoid	Level 2
3	14.321	48,614,400	1.89	2-Coumaranone	C_8_H_6_O_2_	134	553-86-6	2,3-dihydro-1-benzofuran-2-one (or 2(3H)-benzofuranone)	Benzofurans	Level 3
4	14.611	79,126,565	3.08	Benzofuran, 2,3-dihydro-	C_8_H_8_O	120	496-16-2	2,3-dihydro-1-benzofuran	Coumaran	Level 3
5	14.916	51,407,715	2.01	5-Hydroxymethylfurfural	C_6_H_6_O_3_	126	67-47-0	5-(hydroxymethyl)furan-2-carbaldehyde	Carbonyl compounds	Level 2
6	15.722	7,535,482	0.29	Anethole	C_10_H_12_O	148	104-46-1	1-methoxy-4-[(E)-prop-1-enyl]benzene	Anisoles (flavor)	Level 2
7	16.215	6,693,621	0.26	Carvacrol	C_10_H_14_O	150	499-75-2	2-methyl-5-(propan-2-yl)phenol	Monoterpenoids	Level 2
8	17.514	14,603,303	0.56	2,4-Dimethoxyphenol	C_8_H_10_O_3_	154	13330-65-9	2,4-dimethoxyphenol	Methoxyphenols	Level 3
9	20.695	2,608,584	0.11	C-10-Massoia lactone	C_10_H_16_O_2_	168	54814-64-1	6-pentyl-5,6-dihydropyran-2-one	Pyrans	Level 3
10	21.671	19,253,383	0.75	Benzeneacetonitrile, 3-hydroxy-	C_8_H_7_NO	133	25263-44-9	2-(3-hydroxyphenyl)acetonitrile	Benzyl cyanides	Level 3
11	24.757	6,059,254	0.23	6-(Hydroxymethyl)-2,4-dihydro-1,4-benzoxazin-3-one	C_9_H_9_NO_3_	179	615568-17-7	6-(hydroxymethyl)-3,4-dihydro-2H-1,4-benzoxazin-3-one	Benzoxazinones	Level 3
12	27.682	12,416,728	0.48	Tetradecanoic acid	C_14_H_28_O_2_	228	544-63-8	tetradecanoic acid	Fatty Acyls	Level 2
13	29.039	3,136,230	0.12	2-Pentadecanone, 6,10,14-trimethyl-	C_18_H_36_O	268	502-69-2	6,10,14-trimethylpentadecan-2-one	Sesquiterpenoids	Level 3
14	29.415	3,220,645	0.12	2-Hexadecanol	C_16_H_34_O	242	14852-31-4	hexadecan-2-ol	Fatty Acyls	Level 3
15	29.763	4,373,214	0.17	Pentadecanoic acid	C_15_H_30_O_2_	242	1002-84-2	pentadecanoic acid	Fatty Acyls	Level 2
16	29.912	2,945,314	0.11	Hexadecen-1-ol, trans-9-	C_16_H_32_O	240	64437-47-4	(E)-hexadec-9-en-1-ol	Fatty Acyls	Level 3
17	31.367	9,760,387	0.38	Palmitoleic acid	C_16_H_30_O_2_	254	373-49-9	(9Z)-hexadec-9-enoic acid	Fatty Acyls	Level 2
18	32.204	561,504,219	21.87	Palmitic acid	C_16_H_32_O_2_	256	57-10-3	hexadecanoic acid	Fatty Acyls	Level 2
19	33.759	4,869,652	0.18	Margarinic acid	C_17_H_34_O_2_	270	506-12-7	heptadecanoic acid	Fatty Acyls	Level 2
20	34.168	23,933,607	0.93	Heneicosane	C_21_H_44_	296	629-94-7	henicosane	Alkanes	Level 2
21	34.374	6,230,439	0.24	Phytol	C_20_H_40_O	296	150-86-7	(E,7R,11R)-3,7,11,15-tetramethylhexadec-2-en-1-ol	Diterpenoid	Level 2
22	34.847	4,794,573	0.18	Z,Z-10,12-Hexadecadien-1-ol acetate	C_18_H_32_O_2_	280	73829-33-1	(10Z,12Z)-hexadeca-10,12-dien-1-yl acetate	Fatty Acyls	Level 3
23	35.524	1,041,724,274	40.58	cis-Linoleic acid	C_18_H_32_O_2_	280	60-33-3	(9Z,12Z)-octadeca-9,12-dienoic acid	Fatty Acyls	Level 2
24	35.846	87,828,090	3.42	Octadecanoic acid	C_18_H_36_O_2_	284	57-11-4	octadecanoic acid	Fatty Acyls	Level 2
25	36.791	5,281,779	0.21	Linoelaidic acid	C_18_H_32_O_2_	280	506-21-8	(9E,12E)-octadeca-9,12-dienoic acid	Fatty Acyls	Level 2
26	37.787	43,748,849	1.71	Tetracosane	C_24_H_50_	338	646-31-1	tetracosane	Alkanes	Level 2
27	39.158	14,066,999	0.54	Arachic acid	C_20_H_40_O_2_	312	506-30-9	icosanoic acid	Fatty Acyls	Level 2
28	41.115	10,649,548	0.41	Heptacosane	C_27_H_56_	380	593-49-7	heptacosane	Alkanes	Level 2
29	41.499	42,872,963	1.67	2-Monopalmitin	C_19_H_38_O_4_	330	23470-00-0	2-hydroxy-1-(hydroxymethyl)ethyl hexadecanoate	Glycerolipids	Level 2
30	45.03	59,034,816	2.29	2-Methyl-Z,Z-3,13-octadecadienol	C_19_H_36_O	280	519002-96-1	(3Z,13Z)-2-methyloctadeca-3,13-dien-1-ol	Fatty Acyls	Level 3
31	45.738	17,325,089	0.67	α-Monostearin	C_21_H_42_O_4_	358	123-94-4	2,3-dihydroxypropyl octadecanoate	Glycerolipids	Level 2
32	46.03	5,443,478	0.21	4-Hydroxy-10-methyl-3,4,7,8,9,10-hexahydro-2H-oxecin-2-one	C_10_H_16_O_3_	184	56020-71-4	4-hydroxy-10-methyl-3,4,7,8,9,10-hexahydrooxecin-2-one	Oxocins	Level 3
33	47.935	5,688,351	0.22	γ-Sitosterol	C_29_H_50_O	414	83-47-6	(3β,24S)-stigmast-5-en-3-ol	Phytosterol	Level 3
34	49.642	54,395,426	2.12	β-Sitosterol	C_29_H_50_O	414	83-46-5	(3β)-stigmast-5-en-3-ol	Phytosterol	Level 3

Compound identifications are hypothetical; confidence levels (Level 2, 3, or 4 according to MSI norms) are supplied in the ‘Confidence level’ column.

**Table 4:** ZoI (mm), MIC, and MBC values of VACLE.

Bacterium/Dilution	Positive Control	800 μg/mL	400 μg/mL	200 μg/mL	100 μg/mL	MIC μg/mL	MBC μg/mL
*S. aureus*	21 ± 1.45	19 ± 1.31 *	17 ± 0.98 *	14 ± 1.73 *	11 ± 2.45 *	6.25 ± 3.23	12.50 ± 0.22
*E. faecalis*	23 ± 2.45	20 ± 2.11 *	18 ± 2.21 *	14 ± 0.00 *	12 ± 0.74 *	12.50 ± 0.82	25 ± 1.44
*B. subtilis*	19 ± 1.33	17 ± 0.87 *	15 ± 0.76 *	13 ± 0.00 *	11 ± 0.74 *	12.50 ± 0.32	25 ± 0.75
*E. coli*	22 ± 0.43	17 ± 0.56 *	14 ± 0.56 *	12 ± 0.29 *	10 ± 1.82 *	50 ± 0.76	100 ± 1.37
*K. pneumoniae*	20 ± 1.72	17 ± 0.98 *	14 ± 0.85 *	10 ± 0.57 *	8 ± 1.56 *	25.0 ± 1.29	50 ± 0.76
*P. aeruginosa*	23 ± 0.45	16 ± 0.35 *	13 ± 0.34 *	11 ± 1.26 *	9 ± 0.87 *	25.0 ± 1.87	50 ± 0.34

* = *p* < 0.05.

**Table 5:** The ZoI (mm), MIC (μg/mL), and MBC (μg/mL) values of VACSE.

Bacterium/Dilution	Chloramphenicol, 25 µg/mL	800 μg/mL	400 μg/mL	200 μg/mL	100 μg/mL	MIC μg/mL	MBC μg/mL
*S. aureus*	21 ± 1.45	17 ± 1.34 *	15 ± 3.22 *	13 ± 1.45 *	10 ± 2.34 *	12.50 ± 0.25	25. ± 1.77
*E. faecalis*	23 ± 2.45	18 ± 1.23 *	15 ± 3.01 *	12 ± 1.52 *	10 ± 0.32 *	12.50 ± 1.76	25 ± 1.26
*B. subtilis*	19 ± 1.33	16 ± 0.47 *	14 ± 0.73 *	12 ± 0.45 *	10 ± 0.73 *	12.50 ± 0.43	25 ± 0.35
*E. coli*	22 ± 0.43	15 ± 0.33 *	13 ± 0.46 *	11 ± 0.39 *	9 ± 1.42 *	50 ± 1.46	100 ± 1.52
*K. pneumoniae*	20 ± 1.72	16 ± 1.18 *	13 ± 0.45 *	9 ± 0.57 *	6 ± 1.56	100 ± 0.42	50 ± 0.53
*P. aeruginosa*	23 ± 0.45	15 ± 0.42 *	12 ± 1.32 *	10 ± 1.24 *	8 ± 1.81 *	50 ± 0.34	100 ± 0.87

Note: zones of inhibition (ZoI), minimum inhibitory concentration (MIC), minimum bactericidal concentration (MBC). Values are presented as the mean ± SD of triplicate measurements. The results demonstrate a statistically significant decrease from the positive control (chloramphenicol 25 µg/mL), indicated by (* = *p* < 0.05).

**Table 6:** The ZoI (mm), MIC, and MFC values of VACSE against selected *Candida* strains.

*Candida*/Dilution	Fluconazole, 25 µg/mL	800 μg/mL	400 μg/mL	200 μg/mL	100 μg/mL	MIC μg/mL	MFC μg/mL
*C. albicans*	24 ± 2.14	16 ± 1.44 *	14 ± 0.39 *	12 ± 1.35 *	10 ± 2.65 *	50 ± 1.34	100 ± 3.46
*C. glabrata*	23 ± 2.53	18 ± 1.47 *	15 ± 1.52 *	13 ± 1.43 *	11 ± 1.72 *	12.50 ± 1.45	25 ± 1.35
*C. parapsilosis*	22 ± 0.32	19 ± 0.43 *	16 ± 0.34 *	13 ± 1.24 *	12 ± 1.43 *	6.25 ± 2.26	12.50 ± 2.67

* = *p* < 0.05.

**Table 7:** The ZoI, MIC, and MFC values of VACLE against selected *Candida* strains.

*Candida*/Dilution	Fluconazole, 25 µg/mL	800 μg/mL	400 μg/mL	200 μg/mL	100 μg/mL	MIC μg/mL	MFC μg/mL
*C. albicans*	24 ± 2.14	14 ± 1.98 *	12 ± 1.31 *	10 ± 1.37 *	9 ± 0.45 *	50 ± 1.34	100 ± 1.34
*C. glabrata*	23 ± 2.53	15 ± 1.45 *	13 ± 1.32 *	10 ± 1.49 *	9 ± 2.76 *	50 ± 1.34	100 ± 1.28
*C. parapsilosis*	22 ± 0.32	16 ± 2.23 *	13 ± 2.31 *	12 ± 1.14 *	12 ± 0.93 *	25 ± 1.65	50 ± 1.49

Note: minimum inhibitory concentration (MIC), minimum fungicidal concentration (MFC). Values are presented as the mean ± SD of triplicate measurements. The results demonstrate a statistically significant decrease from the positive control, indicated by (* = *p* < 0.05).

## Data Availability

Data is contained within the article.

## Abbreviations

The following abbreviations are used in this manuscript:

GC–MS	Gas Chromatography–Mass Spectrometry
TPC	Total Phenolic Content
TFC	Total Flavonoid Content
DPPH	2,2-Diphenyl-1-picrylhydrazyl
ABTS	2,2′-Azino-bis(3-ethylbenzothiazoline-6-sulfonic acid)
MIC	Minimum Inhibitory Concentration
MBC	Minimum Bactericidal Concentration
